# Tripartite Motif-Containing Protein 22 Interacts with Class II Transactivator and Orchestrates Its Recruitment in Nuclear Bodies Containing TRIM19/PML and Cyclin T1

**DOI:** 10.3389/fimmu.2017.00564

**Published:** 2017-05-15

**Authors:** Greta Forlani, Giovanna Tosi, Filippo Turrini, Guido Poli, Elisa Vicenzi, Roberto S. Accolla

**Affiliations:** ^1^Laboratory of General Pathology and Immunology, Department of Medicine and Surgery, University of Insubria, Varese, Italy; ^2^Viral Pathogens and Biosafety Unit, San Raffaele Scientific Institute, Milano, Italy; ^3^AIDS Immunopathogenesis Unit, San Raffaele Scientific Institute, Milano, Italy; ^4^School of Medicine, Vita-Salute San Raffaele University, Milano, Italy

**Keywords:** HIV-1 restriction factors, tripartite motif-containing protein 22, CIITA, CyclinT1, TRIM19/PML, nuclear bodies

## Abstract

Among interferon (IFN) inducible antiviral factors both tripartite motif-containing protein 22 (TRIM22) and class II transactivator (CIITA) share the capacity of repressing human immunodeficiency virus type 1 (HIV-1) proviral transcription. TRIM22 is constitutively expressed in a subset of U937 cell clones poorly permissive to HIV-1 replication, whereas CIITA has been shown to inhibit virus multiplication in both T lymphocytic and myeloid cells, including poorly HIV-1 permissive U937 cells, by suppressing Tat-mediated transactivation of HIV-1 transcription. Therefore, we tested whether TRIM22 and CIITA could form a nuclear complex potentially endowed with HIV-1 repressive functions. Indeed, we observed that TRIM22, independent of its E3 ubiquitin ligase domain, interacts with CIITA and promotes its recruitment into nuclear bodies. Importantly, TRIM19/promyelocytic leukemia (PML) protein, another repressor of HIV-1 transcription also acting before proviral integration, colocalize in these nuclear bodies upon TRIM22 expression induced by IFN-γ. Finally, tTRIM22 nuclear bodies also contained CyclinT1, a crucial elongation factor of HIV-1 primary transcripts. These findings show that TRIM22 nuclear bodies are a site of recruitment of factors crucial for the regulation of HIV-1 transcription and highlight the potential existence of a concerted action between TRIM22, CIITA, and TRIM19/PML to maintain a state of proviral latency, at least in myeloid cells.

## Introduction

Restriction factors (RF) are specialized host proteins sensing the presence of viral nucleic acids or proteins in order to protect cells from pathogen invasion; they are constitutively expressed prior to virus infection and are usually further inducible by interferons (IFNs) (1). In this regard, human immunodeficiency virus type 1 (HIV-1) infection is counteracted by several RF that target different steps of viral life cycle including capsid uncoating, reverse transcription, nuclear import and integration, proviral transcription, protein translation, virion budding, and release (2). Among these RF, several members of the tripartite motif (TRIM) containing protein are endowed with potent antiviral activity (3, 4). All TRIM proteins are characterized by three highly conserved domains consisting of an amino-terminus Really Interesting New Gene (RING) domain, one or two B-box domains, and a coiled-coil (CC) region, whereas the C-terminal part of the protein varies among family members. The RING domain is characterized by E3 ubiquitin ligase activity, whereas the CC region promotes homo-oligomerization, crucial for the formation of subcellular structures such as nuclear bodies (5).

The first member of the TRIM family that was linked to retroviral restriction has been rhTRIM5α that prevents infection of monkey cells by HIV-1 via targeting its capsid for proteasomal degradation (6). However, human TRIM5α does not exert a similar function in human cells and recent studies suggest that, upon recognition of the capsid lattice, it may act as a trigger of innate immunity via activation of NF-kB and AP-1 transcription factors (7). Another member of the family, TRIM19, also known as promyelocytic leukemia (PML) protein, is induced by IFN (3) and interferes with HIV-1 pre-integration complex (8–10); furthermore, according to recent studies, TRIM19/PML also repressed proviral transcription (11, 12). Finally, tripartite motif-containing protein 22 (TRIM22) has been described to inhibit HIV-1 replication by either affecting virion production (13) if expressed in the cytoplasm, or by suppressing basal as well as phorbol ester-induced HIV-1 long terminal repeat (LTR)-mediated transcription when present in the nucleus (14, 15). In this regard, we have shown that TRIM22 inhibits HIV-1 transcription independent of Tat and NF-kB (15) by interfering with the binding of Specific protein 1 (Sp1) to the HIV-1 LTR promoter region (16). This inhibitory effect of TRIM22 is independent of its E3 ubiquitin ligase activity (15). We also observed that TRIM22 inhibited HIV-1 replication in a subset of U937 promonocytic cell clones poorly permissive to HIV-1 replication (15).

Apart from TRIM proteins, another repressive molecule for HIV-1 expression in human T cells is the class II transactivator (CIITA), originally characterized as a transcriptional activator of major histocompatibility complex class II (MHC-II) genes (17–19), thereby playing a central role in antigen presentation to T lymphocytes. In the context of HIV-1 infection, CIITA inhibits virus replication by competing with the viral transactivator Tat for the binding to the Cyclin T1 subunit of the positive transcription elongation complex (P-TEFb) (20). Subsequently, CIITA inhibits also the replication of the oncogenic human T lymphotrophic virus 1 (HTLV-1) and HTLV-2 by interfering with the HTLV-1 and HTLV-1 Tax-1 and Tax-2 transactivators, respectively (21–25). Thus, CIITA is endowed with a potent dual antiviral activity: it activates the adaptive immune response against pathogens via the regulation of MHC-II genes expression while acting as an endogenous RF against human retroviruses. Recently, we have reported that, like TRIM22, also CIITA was expressed in poorly HIV-1 permissive (*Minus*) U937 cell clones, whereas it was not expressed in HIV-1 permissive (*Plus*) U937 cell clones (26). Interestingly, forced expression of CIITA in Plus U937 clones resulted in the inhibition of Tat-dependent HIV-1 replication independent of TRIM22 (26). Thus, as both CIITA and TRIM22 inhibit HIV-1 transcription and replication, although by different molecular modalities, we investigated whether they could form a complex with the potential to inhibit HIV-1 transcription.

Indeed, we here demonstrate for the first time that TRIM22 binds to CIITA independent of its own E3 ubiquitin ligase activity; this interaction leads to the formation of nuclear bodies containing CIITA and Cyclin T1. Furthermore, upon induction of TRIM22 expression by IFN-γ stimulation, endogenous TRIM19/PML, previously described to be present in subnuclear structures known as PML nuclear bodies (PML-NBs) (27), was recruited in such nuclear bodies. Overall, these observations suggest that TRIM22 promotes the assembly of several RF in specific nuclear compartments acting either before or after proviral integration. Altogether these results strongly suggest that different host RF may act in concert to interfere with discrete steps of the retroviral life cycle.

## Materials and Methods

### Plasmids

Plasmid expressing myc epitope-tagged CIITA full-length (1-1130) (pcmCIITA) vector was previously described (28). Plasmids expressing flag epitope-tagged TRIM22 and flag epitope-tagged deltaRING TRIM22 (ΔRING TRIM22) were previously described (15). pHA-Cyclin T1 vector was a gift from M. B. Peterlin (UCSF).

### Cell Cultures

Human embryonic kidney 293T cells (kindly provided by Prof. B.M. Peterlin, UCSF, San Francisco, CA, USA) and Human squamous carcinoma Hep-2 cells (kindly provided by Dr. F. Bex, Universitè Libre de Bruxelles, Brussels, Belgium) were cultured in Dulbecco’s modified Eagle medium containing 5 mM l-glutamine and supplemented with 10% fetal calf serum.

### Antibodies and Reagents

The following antibodies were used for western blot analysis: mouse anti-FLAG M2 (Sigma-Aldrich, F1804, diluted 1:6,000); mouse anti-CIITA (7-1H, 1:1,000; Santa Cruz Biotechnology). For immunofluorescence staining, the following antibodies were used: fluorescein isothiocyanate (FITC)-conjugated mouse anti-FLAG-M2 (Sigma-Aldrich, F4049, diluted 1:200); mouse anti-HA (Sigma-Aldrich, H9658, diluted 1:400); mouse anti-PML (Santa Cruz Biotechnology, sc-966, diluted 1:200); rabbit anti-PML (Santa Cruz Biotechnology, sc-5621, diluted 1:200); rabbit anti-myc (Santa Cruz Biotechnology, sc-789, diluted 1:200); mouse anti-myc (Santa Cruz Biotechnology, sc-40, diluted 1:200); mouse anti-TRIM22 (Sigma-Aldrich, SAB14069, diluted 1:200); rabbit anti-CCNT1 (Sigma-Aldrich, HPA004892, diluted 1:200). The secondary antibodies (Life Technology) used for immunofluorescence and confocal analyses are specified in the figure legends.

Reagents used in this study were human IFN-γ (Origene, TP721239), ANTI-FLAG M2 Affinity agarose Gel (Sigma-Aldrich, A2220), Arsenic (III) oxide (Sigma-Aldrich, 202673), and FluorSave reagent (Calbiochem, 345789).

### Transient Transfection, Luciferase Assay

A total of 293T cells were seeded in 96-well plates and co-transfected with a Luciferase (Luc)-expressing plasmid (20 ng) under the control of HIV-1 LTR (kindly donated by Nadir Mechti, Montpellier, France), in combination with 200 ng of pc-TRIM22-expressing plasmid or empty pcDNA3.1 as a control. Twenty-four hours post-transfection, cells were incubated with arsenic trioxide (As_2_O_3_) (1 µM), a known inhibitor of TRIM19/PML (11); the Luc activity assay (Promega) was performed 24 h after incubation.

### Immunoprecipitation

For protein binding studies, 293T cells were seeded on 100 mm-diameter petri dishes and transfected with pcmCIITA (2 µg) alone or in combination with 2 µg of flag-TRIM22 or flag-ΔRING by using FugeneHD (Promega). Empty pcDNA3 vector was used as a stuffer DNA. Twenty-four hours after transfection, the cells were lysed on ice for 45 min with lysis buffer (1% NP-40, 10 mM Tris–HCl pH 7.4, 150 mM NaCl, 2 mM EDTA) supplemented with 0.1% protease inhibitor cocktail (Sigma-Aldrich). Pre-cleared cell lysates were immunoprecipitated (IP) overnight at 4°C with the anti-FLAG M2 affinity agarose gel (Sigma-Aldrich). Precipitated proteins were resolved on 8% SDS-PAGE (polyacrylamide gel electrophoresis) and analyzed by immunoblotting with anti-CIITA and anti-FLAG M2 antibodies. Ten percent of total cell extract was used to detect protein expression by Western blotting (input).

### Immunofluorescence and Confocal Microscopy

For localization studies, Hep-2 cells cultured on glass cover slips were transfected with 0.2 µg of each of the following plasmids: pcmCIITA, pfTRIM22, pfΔRING, and pHA-Cyclin T1. At 24 h post-transfection, cells were processed as previously described (29). Cells were stained overnight with the specific primary antibodies followed by the appropriate secondary antibodies as indicated in the figure legends. For flag-tagged TRIM22 staining, the cells were stained with FITC-conjugated anti-flag antibody (1:200 dilution in 1× PBS, 0.1% BSA) for 2 h at RT in the dark. After extensive washings with 1× PBS, the slides were mounted on cover-slips by using the FluorSave reagent (Calbiochem) and examined by a confocal laser-scanning microscope (Leica TCS SP5; objective lenses: HCX PL APO, 63× original magnification, numerical aperture 1.25). Images were acquired and analyzed by LAS AF software.

## Results

### TRIM22 Interacts with CIITA *In Vivo*

**Figure 1:**
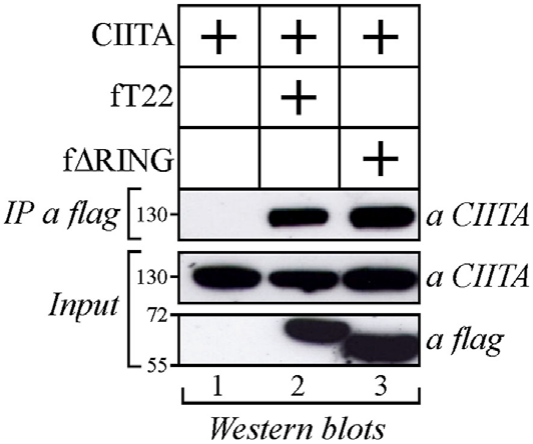
**Class II transactivator (CIITA) interacts with tripartite motif-containing protein 22 (TRIM22) independent from the Really Interesting New Gene (RING) domain**. 293T cells were transfected with plasmid coding for myc-tagged CIITA alone or in combination with vectors expressing flag-tagged TRIM22 full-length (fT22) or TRIM22 RING mutant (fΔRING). Cell extracts were immunoprecipitated (IP) with anti-flag monoclonal antibody (mAb) (IP a flag) and the IP complexes were analyzed by western blotting with anti-CIITA mAb. Ten percent of the whole cell extract was analyzed by Western blotting for the expression of TRIM22 full-length, its deletion mutant TRIM22-ΔRING and CIITA (input).

As both TRIM22 and CIITA act as independent repressors of HIV-1 transcription, we first investigated whether they could associate. Flag epitope-tagged TRIM22 (fT22) and Myc epitope-tagged CIITA (CIITA) were transiently expressed in 293T cells. Cell lysates were IP with an anti-flag monoclonal antibody (mAb) and TRIM22-bound proteins were assessed for the presence of CIITA by Western blotting with an anti-CIITA mAb. Indeed, CIITA co-precipitated with TRIM22 (Figure 1, lane 2) and this interaction was specific in that CIITA was not detected in the absence of TRIM22 (Figure 1, lane 1). CIITA-TRIM22 binding occurred independent of the TRIM22 RING domain, as ΔRING mutant (fΔRING) still co-precipitated with CIITA (Figure 1, lane 3).

### CIITA Is Recruited in TRIM22-Containing Bodies both in the Nucleus and in the Cytoplasm

**Figure 2:**
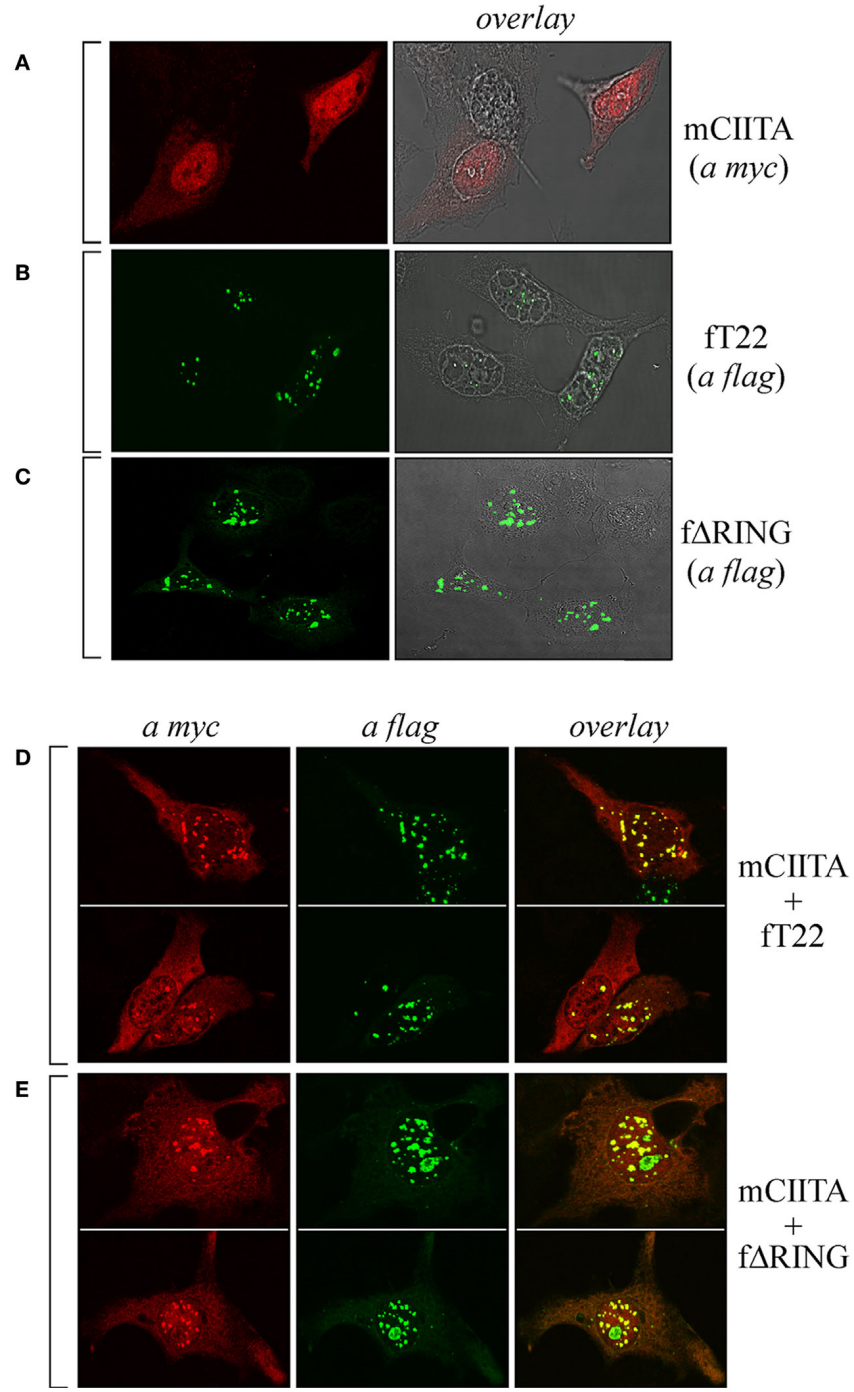
**The subcellular distribution of class II transactivator (CIITA) is affected by tripartite motif-containing protein 22 (TRIM22)**. Hep-2 cells were transfected with either myc-tagged CIITA (mCIITA) **(A)**, flag epitope-tagged TRIM22 full-length (fT22) **(B)**, or flag epitope-tagged TRIM22-ΔRING (fΔRING) **(C)** vectors alone. **(A–C)** The overlay panels were obtained by merging the differential interference contrast image with the corresponding immunofluorescence image. mCIITA was co-transfected in Hep-2 cells either with fT22 **(D)** or fΔRING **(E)**, as specified on the right of the panels. Cells were fixed, first stained with anti-myc rabbit polyclonal antibody followed by goat anti-rabbit AlexaFluor 546-conjugated antibody to detect myc CIITA and then stained with fluorescein isothiocyanate-conjugated anti-flag antibody to detect flag-TRIM22 (fT22) or flag-ΔRING deletion mutant. Each panel shows at least two representative fields. **(A–E)** Stained cells were then analyzed by confocal microscopy, as described in Section “Materials and Methods.”

As CIITA and TRIM22 can interact, we next tested whether they colocalize in specific subcellular compartments. To this aim, Hep-2 cells, which do not constitutively express either CIITA or TRIM22, were transiently transfected with either a flag-tagged full-length TRIM22 (fT22), with its ΔRING mutant (fΔRING) or with myc-tagged CIITA (mCIITA). Their cellular distribution was assessed by both immunofluorescence and confocal microscopy. While CIITA exhibited a predominant nuclear accumulation and a diffused, less abundant cytoplasmic distribution (Figure 2A), TRIM22, and its ΔRING mutant were mostly localized in nuclear bodies and in few punctuated structures in the cytoplasm (Figures 2B,C, respectively).

Upon cell co-transfection with both plasmids, expression of TRIM22 led to recruitment of a fraction of CIITA into nuclear bodies (Figure 2D, overlay). This effect was independent of TRIM22 RING domain as the cells transfected with TRIM22 ΔRING mutant showed the same localization pattern of CIITA in nuclear bodies (Figure 2E, overlay). These results, together with the co-IP findings, demonstrate that TRIM22 and CIITA associate in specific subcellular structures mostly localized in the nucleus.

### Endogenous TRIM22 Significantly Colocalizes with PML in Nuclear Bodies and Nuclear CIITA Colocalizes with TRIM22/PML-Containing Nuclear Bodies

**Figure 3:**
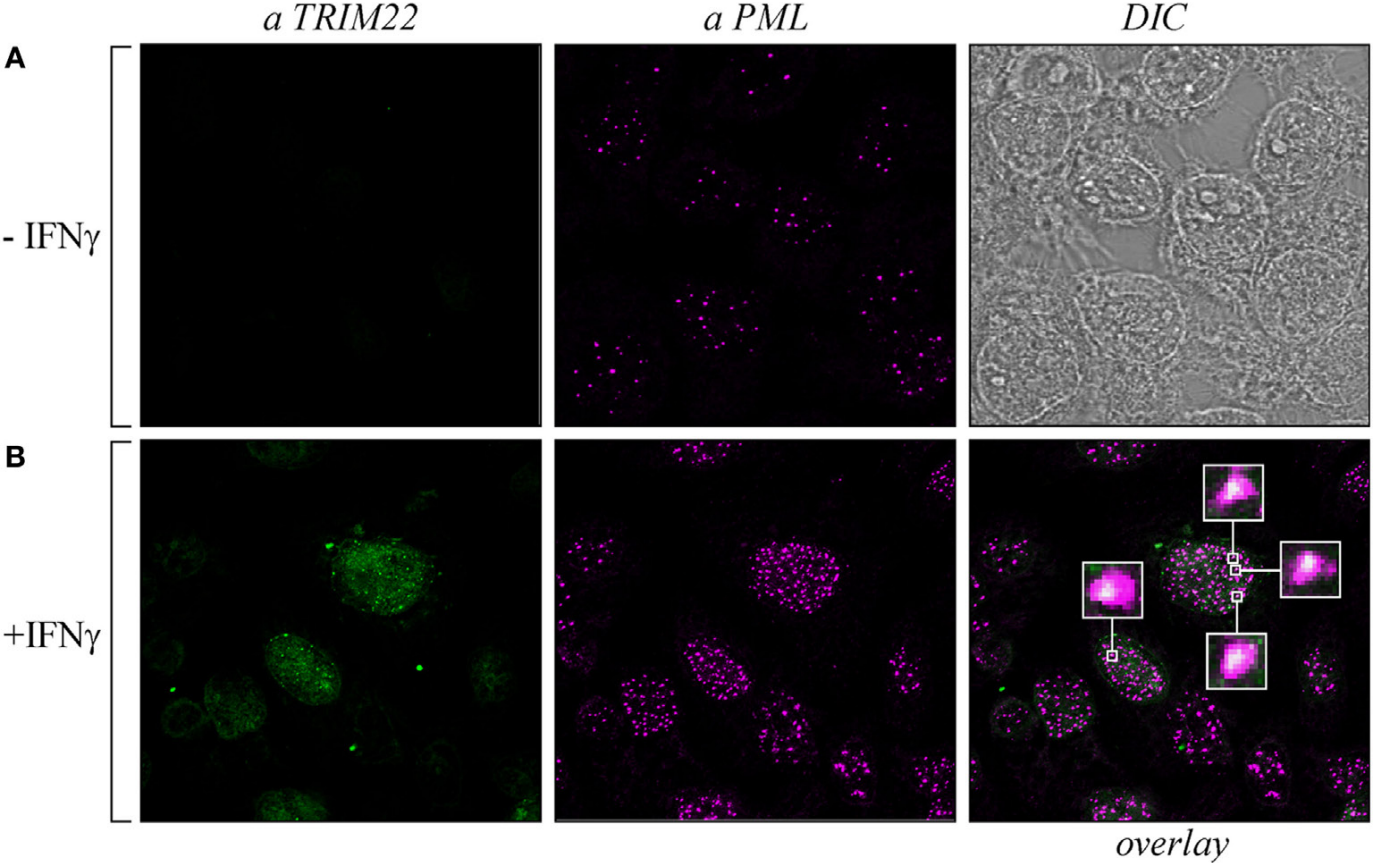
**Promyelocytic leukemia (PML) colocalized with endogenous tripartite motif-containing protein 22 (TRIM22) in Hep-2 cells treated with IFNγ**. Hep-2 cells were incubated with IFN-γ (150 U/ml) (+IFNγ) **(B)** or with vehicle (−IFNγ) **(A)** for 48 h and analyzed by immunofluorescence and confocal microscopy. TRIM22 was detected with anti-TRIM22 mouse monoclonal antibody followed by goat anti-mouse AlexaFluor 633-conjugated antibody. PML was detected with anti-PML rabbit polyclonal antibody followed by goat anti-rabbit AlexaFluor 546-conjugated antibody. Insets show enlarged views of the areas indicated by the white squares. DIC is the differential interference contrast image.

**Figure 4:**
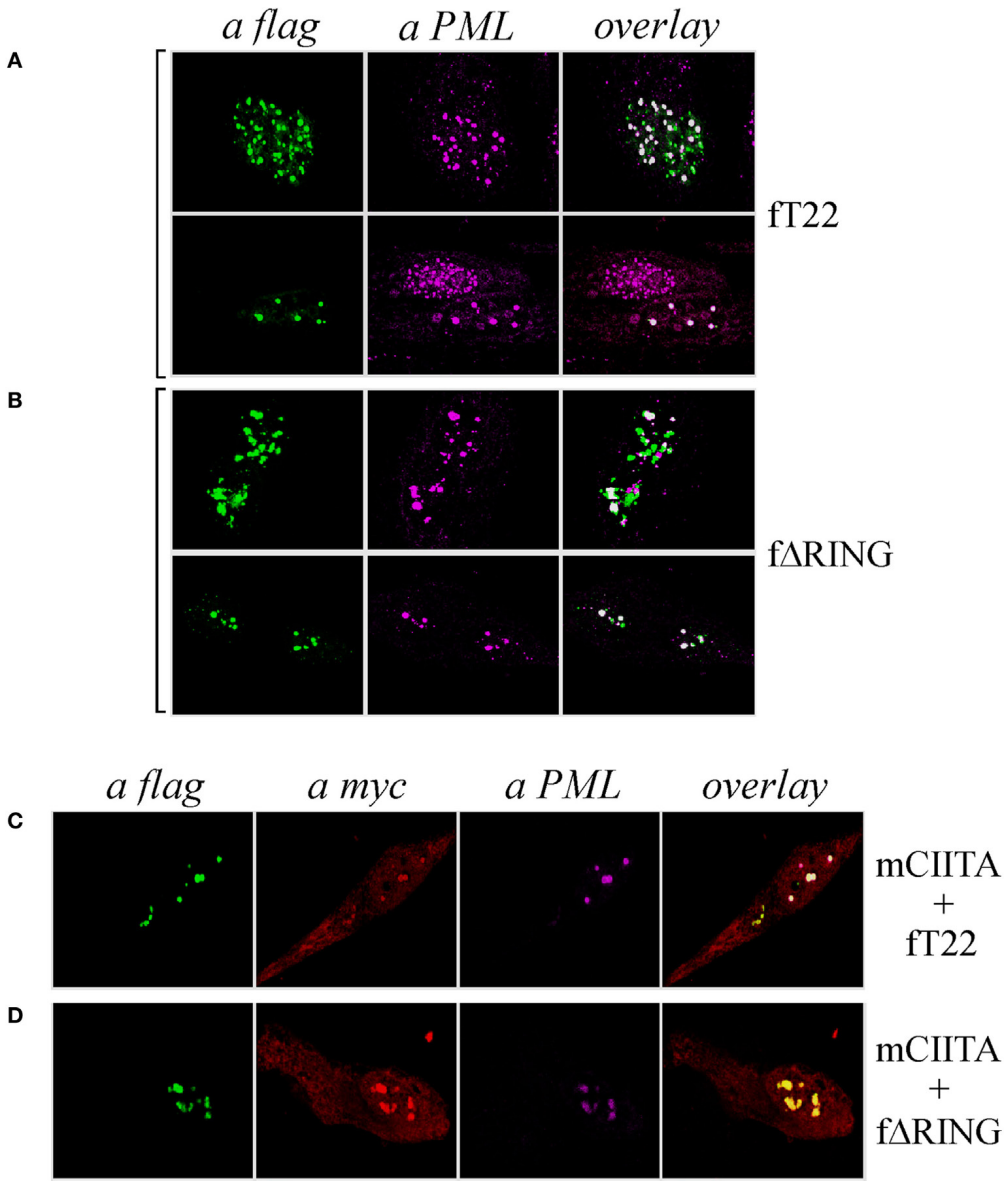
**Class II transactivator (CIITA) colocalizes with tripartite motif-containing protein 22 (TRIM22) and promyelocytic leukemia (PML) in nuclear bodies**. Hep-2 cells were transfected with flag-tagged TRIM22 (fT22) **(A)** or with flag-tagged TRIM22-ΔRING (fΔRING) **(B)** expression vectors, incubated with the antibodies listed on the top and analyzed by immunofluorescence and confocal microscopy. Each panel shows two representative fields. Endogenous PML was detected by using the anti-PML mouse monoclonal antibody followed by goat anti-mouse AlexaFluor 546-conjugated antibody. Flag-TRIM22 and flag-ΔRING were detected by direct staining with fluorescein isothiocyanate-conjugated anti-flag antibody. Confocal microscopy analysis was carried out in Hep-2 cells transfected with fTRIM22 **(C)** or with fΔRING **(D)** expression vectors in the presence of myc-tagged CIITA plasmid, as indicated on the right of the panels. CIITA protein was detected by using the anti-myc rabbit polyclonal antibody followed by goat anti-rabbit AlexaFluor 647-conjugated antibody. Endogenous PML protein was detected by using the anti-PML mouse monoclonal antibody followed by goat anti-mouse AlexaFluor 546-conjugated antibody. TRIM22 and TRIM22-ΔRING were detected as in **(A, B)**.

As both TRIM19/PML and TRIM22 are IFN-inducible genes (30), we tested whether IFN-γ stimulation affected PML-NB and TRIM22 distribution. To this purpose, Hep-2 cells, which constitutively express endogenous TRIM19/PML, but not TRIM22 (Figure 3A, αPML and αTRIM22, respectively), were stimulated with IFN-γ for 48 h and were then analyzed for TRIM22 and TRIM19/PML distribution. IFN-γ stimulation induced the expression of TRIM22 that assumed a characteristic nuclear dot distribution (Figure 3B, αTRIM22), while the number of PML dotted-like structures increased substantially (Figure 3B vs Figure 3A, αPML). Remarkably, a significant number of TRIM22-specific dots colocalized with those containing endogenous TRIM19/PML (Figure 3B, overlay). Upon overexpression of TRIM22 by transfection, specific nuclear dots were more defined and apparent (Figure 4A, αflag) and, interestingly, the expression pattern of endogenous TRIM19/PML in TRIM22-overexpressing cells changed significantly from a multi-microdot pattern to fewer and larger dots containing TRIM22 (Figure 4A, αPML and overlay); similar findings were observed after transfection with TRIM22 ΔRING mutant (Figure 4B, αflag) with respect to endogenous TRIM19/PML (Figure 4B, αPML and overlay), indicating that the RING domain is dispensable for the co-localization of TRIM22 with TRIM19/PML. These results represent the first demonstration that endogenous TRIM22 colocalizes with another TRIM protein, i.e., endogenous TRIM19/PML.

Previous studies showed that CIITA localized in TRIM19/PML bodies in Hep-2 cells stimulated with IFN-γ and that this localization protected CIITA from proteasomal degradation (31). Therefore, we tested whether CIITA colocalized in nuclear bodies together with TRIM22 and TRIM19/PML in Hep-2 cells. Indeed, co-expression of CIITA (Figures 4C,D, αmyc) resulted in co-localization of the protein in the same nuclear dots containing TRIM22 and PML (Figure 4C, overlay), or ΔRING and PML (Figure 4D, overlay).

### The Recruitment of PML in TRIM22-Containing Nuclear Bodies Does Not Prevent Arsenic-Dependent PML Degradation

**Figure 5:**
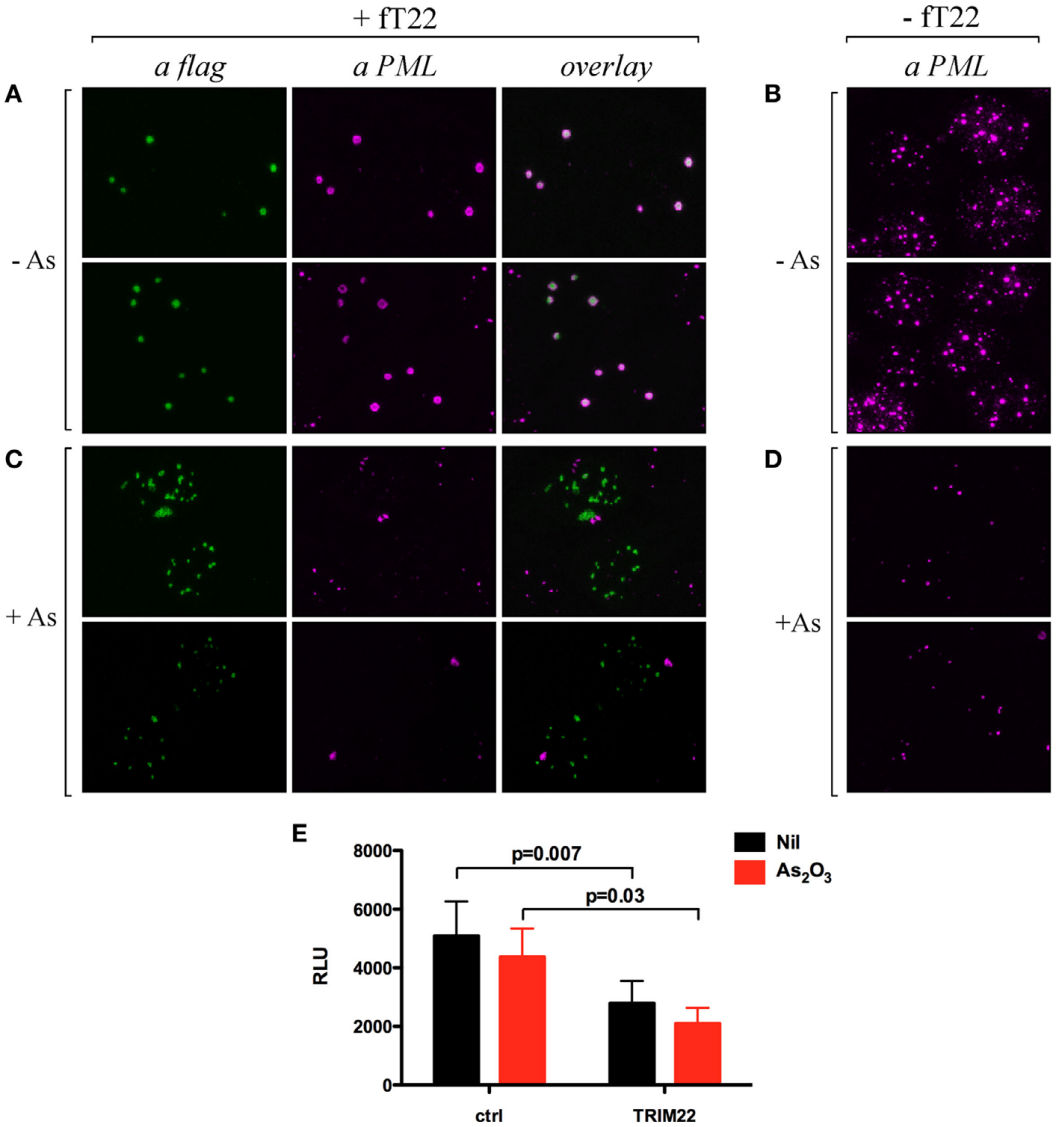
**Effect of arsenic treatment on tripartite motif-containing protein 22 (TRIM22) and promyelocytic leukemia (PML) cellular distribution in Hep-2 cells**. Hep-2 cells transfected with fTRIM22 **(A,C)** or with empty vectors **(B,D)** were exposed to 1 mM arsenic trioxide (+As) **(C,D)** or to vehicle (−As) **(A,B)** for 8 h. Cells were analyzed by immunofluorescence and confocal microscopy by using the anti-PML mouse monoclonal antibody followed by goat anti-mouse AlexaFluor 546-conjugated antibody. Flag-TRIM22 was detected by direct staining with fluorescein isothiocyanate-conjugated anti-flag antibody. **(E)** 293T cells were transfected with a Luciferase reporter under the control of HIV-LTR and a TRIM22 expressing plasmid in a ratio 1:10, respectively, with and without As_2_O_3_ treatment (1 µM). The Luciferase activity was determined 24 h post-treatment. RLU indicates Relative Luciferase Unit. Bars indicate the mean ± SEM of two independent experiments in triplicates. *P* value was calculated by a paired *t* test.

Cell incubation with arsenic trioxide (As_2_O_3_) results in posttranslational modification of TRIM19/PML and its consequent degradation by the proteasome (32, 33). Thus, we verified whether the recruitment of TRIM19/PML in TRIM22-containing nuclear bodies prevented its As_2_O_3_-induced degradation. Hep-2 cells transfected with flag-tagged TRIM22 expression vector (+fT22) or with the empty plasmid (−fT22) were exposed to As_2_O_3_ for 8 h (or to vehicle) and were then analyzed by immunofluorescence and confocal microscopy. In the absence of As_2_O_3_, TRIM19/PML localized into PML-NBs (Figure 5B), whereas, as shown before, it accumulated in large nuclear structures corresponding to TRIM22-containing bodies in cells expressing TRIM22 (Figure 5A, αPML and overlay). In contrast, upon incubation with As_2_O_3_, a dramatic reduction in PML fluorescence was observed (Figure 5D), confirming previous findings (34). Similar results were obtained for Hep-2 cells expressing TRIM22 (Figure 5C, αPML), indicating that TRIM19/PML interaction with TRIM22 does not prevent As_2_O_3_-mediated PML degradation. Importantly, TRIM22 subcellular distribution was not affected by As_2_O_3_ (Figure 5C vs Figure 5A, αflag).

Next, we assessed the ability of TRIM22 to inhibit basal HIV-1 LTR transcription in the presence of As_2_O_3_ by measuring the HIV-1-LTR luciferase activity in 293T cells. Consistent with its lack of effect on TRIM22 expression and localization, As_2_O_3_ did not induce basal HIV-1 LTR transcription, and more importantly, did not affect the ability of TRIM22 to suppress basal transcription from HIV-1 LTR promoter (Figure 5E). Thus, TRIM22 recruited TRIM19/PML in specific nuclear structures without either preventing arsenic-mediated PML degradation or being affected by the poison in terms of its own nuclear localization or repressive transcriptional activity.

### Endogenous Cyclin T1 Is Recruited in CIITA-TRIM22-Containing Nuclear Bodies

**Figure 6:**
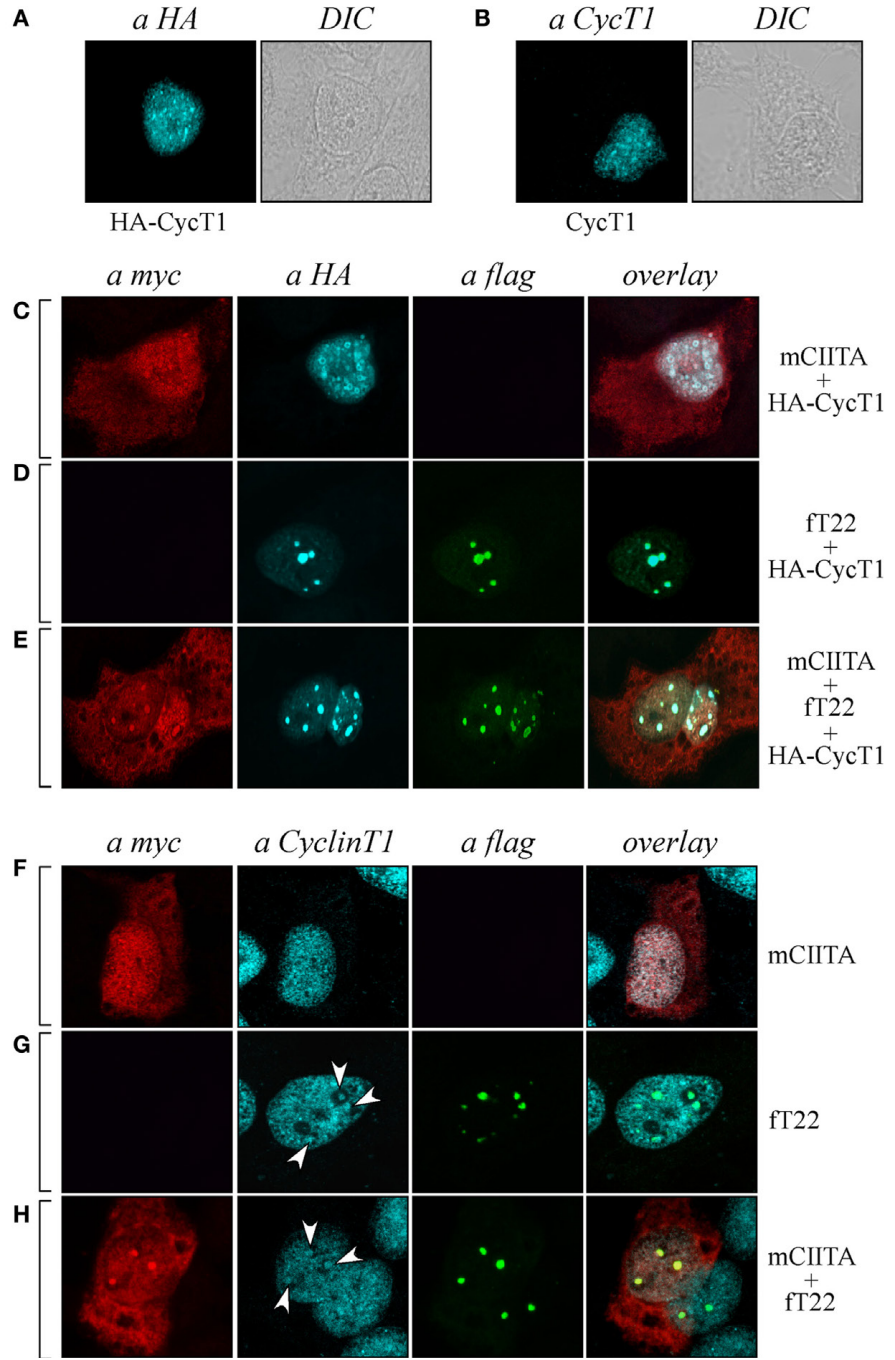
**Tripartite motif-containing protein 22 (TRIM22) recruits both class II transactivator (CIITA) and Cyclin T1 in nuclear bodies**. **(A)** The expression of hemagglutinin (HA)-tagged Cyclin T1 (HA-CycT1) or **(B)** endogenous Cyclin T1 was assessed in Hep-2 cells incubated with a mouse monoclonal anti-HA antibody followed by a goat anti-mouse AlexaFluor 633-conjugated antibody or with a rabbit polyclonal anti-Cyclin T1 antibody followed by goat anti-rabbit AlexaFluor 546-conjugated antibody, respectively, and analyzed by immunofluorescence and confocal microscopy analysis. DIC images are shown (right squares). **(C,D,E)** Hep-2 cells were transfected with mCIITA, HA-CycT1, and TRIM22 (fT22) expression vectors in different combinations, as indicated on the right, and incubated with the antibodies listed on the top. Anti-myc and anti-HA staining was detected with goat anti-rabbit AlexaFluor 546-conjugated and goat anti-mouse AlexaFluor 633-conjugated antibodies, respectively. Flag-TRIM22 was detected by direct staining with fluorescein isothiocyanate-conjugated anti-flag antibody. **(F,G,H)** Hep-2 cells were transfected with mCIITA and TRIM22 (fT22) expression vectors in different combinations. Anti-myc and anti-Cyclin T1 staining were detected with goat anti-mouse AlexaFluor 633-conjugated and goat-anti-rabbit AlexaFluor 546-conjugated antibodies, respectively. Flag-TRIM22 was detected as in **(D,E)**.

As CIITA inhibits Tat transcriptional function by competing with Tat for the binding to Cyclin T1 subunit of the P-TEFb complex (20), we tested whether Cyclin T1 was recruited in CIITA-TRIM22-containing nuclear bodies. Hemagglutinin (HA) epitope-tagged Cyclin T1 (HA-CyclinT1) transfected in Hep-2 cells localized in the nucleus in a speckle-like pattern (Figure 6A, αHA), as previously shown in other cell lines (33). Since the nuclear distribution pattern of endogenous Cyclin T1 (Figure 6B), αCyclinT1 is similar to that observed for the overexpressed protein (Figure 6A), we first tested whether CIITA colocalized with the overexpressed Cyclin T1. By co-transfecting CIITA and Cyclin T1, we observed that Cyclin T1 localized in numerous nuclear dots (Figure 6C), αHA, most of which were overlapping with those containing CIITA (Figure 6C, overlay). The distribution of CIITA is not affected by the expression of CyclinT1 (Figure 6C, αmyc). Surprisingly, the expression of TRIM22 changed dramatically the nuclear distribution of Cyclin T1. Indeed accumulated in peculiar large TRIM22-containing nuclear dots (Figure 6D, αHA and overlay). When TRIM22, Cyclin T1, and CIITA were co-expressed simultaneously in the cells, the distribution pattern of Cyclin T1 and TRIM22 was not affected by CIITA (Figure 6E, αHA and αflag, respectively), whereas, instead, a significant amount of nuclear CIITA was found to colocalize in the same nuclear bodies containing Cyclin T1 and TRIM22 (Figure 6E, overlay).

In order to assess whether the distribution of endogenous Cyclin T1 (Figure 6B) was modified in the presence of CIITA and TRIM22 in a similar fashion as described in Cyclin T1-overexpressing cells, Hep-2 cells transfected with CIITA, TRIM22, or with both CIITA and TRIM22 were analyzed by immunofluorescence and confocal microscopy. In presence of CIITA, Cyclin T1 showed a nuclear pattern similar to that observed with the overexpressed protein, with small speckle-like diffuse distribution in the nucleus (Figure 6F, αCyclinT1) largely overlapping with nuclear CIITA (Figure 6F, overlay). In presence of TRIM22, endogenous Cyclin T1 partially accumulated in few larger nuclear bodies (Figure 6G, αCyclinT1, white arrows), corresponding to the TRIM22-containing nuclear bodies (Figure 6G, overlay). The concomitant overexpression of CIITA did not alter the nuclear accumulation of Cyclin T1 in TRIM22 nuclear bodies (Figure 6H, αCyclinT1, white arrows, and αflag, respectively) and again, nuclear CIITA was recruited in the same nuclear bodies containing Cyclin T1 and TRIM22 (Figure 6H, overlay), thus confirming the results observed with the exogenous Cyclin T1.

Taken together, these results indicate that Cyclin T1 and nuclear CIITA significantly colocalize in dot-like structures and, in presence of TRIM22, they are recruited in larger TRIM22-containing nuclear bodies.

### TRIM22 Induces the Localization of PML and Cyclin T1 in Specific Subnuclear Structures

**Figure 7:**
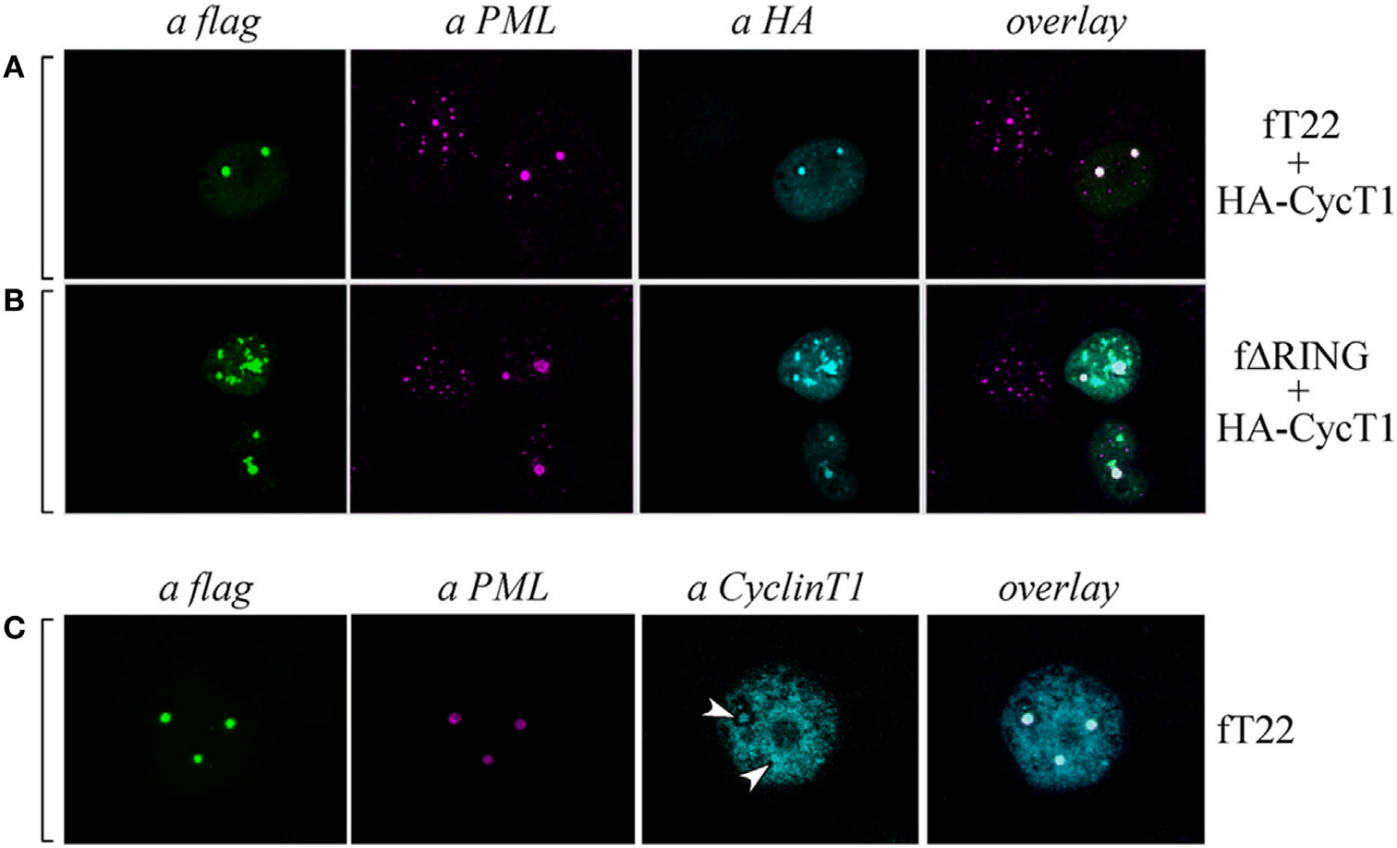
**Cyclin T1 and promyelocytic leukemia (PML) are recruited in tripartite motif-containing protein 22 (TRIM22)-containing nuclear bodies**. **(A,B)** Hep-2 cells were transfected with hemagglutinin (HA)-tagged CycT1, with flag-tagged TRIM22 (fT22) or with flag-tagged TRIM22-ΔRING (fΔRING) expression vectors as indicated on the right, and incubated with the antibodies listed on the top. PML was detected with anti-PML rabbit polyclonal antibody followed by AlexaFluor 546-conjugated antibody. Cyclin T1 was detected with anti-HA monoclonal antibody (mAb) followed by AlexaFluor 633-conjugated antibody. Flag-TRIM22 and flag ΔRING were detected by direct staining with fluorescein isothiocyanate (FITC)-conjugated anti-flag antibody. **(C)** Hep-2 cells were transfected with with flag-tagged TRIM22 (fT22) expression vector as indicated on the right, and incubated with the antibodies listed on the top. PML was detected with anti-PML mouse mAb followed by AlexaFluor 546-conjugated antibody. Cyclin T1 was detected with anti-Cyclin T1 rabbit polyclonal antibody followed by AlexaFluor 647-conjugated antibody. Flag-TRIM22 was detected by direct staining with FITC-conjugated anti-flag antibody.

As Cyclin T1 interacts with the PML protein within specific subnuclear compartments that are coincident with PML-NBs and, after Tat-mediated HIV-1 LTR transactivation, both PML and CyclinT1 are recruited to the viral promoter at the periphery of the bodies (35), we tested whether also PML colocalized in the same nuclear bodies containing both TRIM22 and CyclinT1. Hep-2 cells were transfected with TRIM22 and CyclinT1 expression vectors and their expression and localization, together with that of endogenous PML, was assessed by immunofluorescence and confocal microscopy. As observed in cells overexpressing TRIM22 (Figure 7A, αflag), endogenous PML localized in large dots corresponding to TRIM22 nuclear bodies containing also CyclinT1 (Figure 7A, overlay). The same findings were observed with the ΔRING mutant (Figure 7B, overlay) indicating that the absence of the RING domain does not affect the recruitment of both PML and CyclinT1 in TRIM22 nuclear bodies. Localization of Cyclin T1 in PML-containing TRIM22 nuclear bodies was not an artifact of transfection as endogenous Cyclin T1 partially colocalized with this nuclear structures (Figure 7C, αCyclinT1, white arrows and overlay).

These findings together with the above results showing that CIITA localizes in TRIM22 nuclear bodies containing PML (Figure 4C) and Cyclin T1 (Figures 6E,H), demonstrate that all four factors can reside in the same nuclear bodies whose formation is driven by TRIM22, and strongly suggest that the driving force promoting and/or facilitating the recruitment of the other three factors in the same subnuclear compartments.

## Discussion

Several RF have been described as capable of interfering with distinct steps of the HIV-1 life cycle either before or after integration of the provirus into host DNA. We have here investigated the hypothesis that two of such RF, TRIM22 and CIITA, acting as independent negative regulators of proviral transcription and co-regulated in *Minus* U937 cell clones (15, 16, 20, 26), could physically interact and form molecular complexes either in the cell cytoplasm or nucleus. We here indeed show that TRIM22 interacts with CIITA leading to the recruitment of CIITA into TRIM22-containing nuclear bodies. Furthermore, upon cell stimulation with IFN-γ, TRIM22 nuclear bodies were found to contain TRIM19/PML (another HIV-1 RF acting on the pre-integration complex and also as a negative regulator of proviral transcription). In addition in the TRIM22 bodies, we also found CyclinT1, a crucial component of P-TEFb required for the elongation of HIV-1 primary transcripts (36).

Despite the knowledge acquired on the molecular nature and corresponding mechanisms of action of the different cellular RF that counteract viral infection, replication and spreading, particularly of HIV-1, which factors control their cellular sub-localization (i.e., cytoplasmic vs. nuclear) and eventually coordinate their activities during infection are essentially unknown (37, 38). The reason to investigate in detail the possible functional and biochemical interaction between TRIM22 and CIITA stemmed from our recent observation that these two factors are both expressed in poorly HIV-1 permissive, *Minus* U937 myeloid cell clones, but not in their counterpart, i.e., HIV-1 permissive U937 *Plus* cell clones (26). When TRIM22 or CIITA were individually overexpressed in *Plus* U937 cells they similarly suppressed HIV-1 replication without reaching, however, the inhibition level observed in the U937 *Minus* cells (26). These findings suggested that the simultaneous expression of these two RF resulted in a more effective HIV-1 restriction (26). Indeed, the results reported in the present study, although obtained in a setting of overexpression of TRIM22 and CIITA and thus in a not completely physiological conditions, clearly show that the two RF can associate in specific, speckle-like nuclear structures. Interestingly, the recruitment of nuclear CIITA into the TRIM22 nuclear bodies indicated that TRIM22 plays a leading role in the co-localization of the two RF. The prevalent nuclear distribution of TRIM22 in a speckle-like pattern has been already observed by other investigators (39–42). In some cells, TRIM22 localizes in nuclear bodies partially overlapping with Cajal bodies (40) or with centrosome (43). The C-terminal B30.2/SPRY domain was shown to be required for the nuclear localization and formation of nuclear bodies (39, 40). TRIM22 was also found in the cytoplasm associated with vimentin-containing structures (43). These variations in the subcellular distribution of TRIM22 may however depend on the cell type, the cell cycle, the epitope tag, and the method of fixation used in the analysis (5).

Tripartite motif-containing protein 22, as most TRIM family members, is induced by IFN-γ, a functional feature shared with TRIM19, also known as PML protein. Hep-2 cells express constitutive levels of TRIM19/PML, but not of TRIM22. However, IFN-γ stimulation of these cells leads to the prompt induction of TRIM22 as well as to the upregulation of TRIM19/PML. Interestingly, IFN-induced TRIM22 localized in speckle-like structure similar to the TRIM22-containing nuclear bodies observed in TRIM22-transfected cells and, of note, a significant number of these nuclear bodies contained also endogenous TRIM19/PML. Furthermore, CIITA was recruited in these nuclear bodies containing also PML in cells co-expressing TRIM22 and TRIM19/PML.

We believe that these findings are potentially relevant for several reasons. First, they show for the first time that TRIM22 and TRIM19/PML, two RF strongly implicated in the natural immunity against HIV-1 and other viruses, can localize in the same nuclear structures. Whether these nuclear structures overlap in content and function with PML-NBs, known to be a sort of hub of intense biological activity, protein–protein interactions, nuclear storage and/or sequestration of proteins, and posttranslational modification of proteins (44), was not assessed here and will be the focus of future investigation. However, TRIM22 expression failed to prevent arsenic-induced TRIM19/PML degradation, suggesting that this RF did not undergo significant modifications within these nuclear structures. As arsenic induces a poly-sumoylation of TRIM19/PML and this posttranslational modification is needed for its degradation by the proteasome (33), our results suggest that the localization of PML in TRIM22 bodies does not affect its binding to SUMO. Conversely, TRIM22 expression was not affected by arsenic with TRIM22 maintaining its inhibitory effect on basal HIV-1 transcription. As it has been previously observed that in Hep-2 cells CIITA may localize in PML-NBs where it is protected from proteasomal degradation (31), we can speculate that TRIM22-containing nuclear bodies may exert a similar function.

An additional potentially relevant observation of our study is that Cyclin T1, a crucial component of the P-TEFb complex, also colocalized with CIITA, TRIM22, and TRIM19/PML in TRIM22-containing nuclear bodies. In this regard, Marcello et al. have previously observed that Cyclin T1 physically interacts with PML protein and accumulates in nuclear PML bodies. Furthermore, this association was suggested to negatively regulate Tat-mediated HIV-1 LTR transcription by modulating the availability of P-TEFb to the cell transcriptional elongation machinery (35). Thus, it is tempting to speculate that CIITA may synergize with the action of TRIM19/PML and further inhibit Tat-mediated HIV-1 LTR transactivation by competing with Tat for the binding to Cyclin T1, as we have previously reported in human T cells (20). Furthermore, P-TEFb also drives NF-kB-dependent HIV-1 LTR transcription (45). Thus, it is likely that TRIM22, by segregating CyclinT1 into the nuclear bodies, could also affect indirectly NF-kB-induced viral transcription. At this level, a potential synergy among CIITA, TRIM19/PML, and TRIM22 can be hypothesized to affect either Sp1-dependent and/or Tat-driven transcription of HIV-1. Worthy of note, TRIM22, although not physically interacting with Sp1, interferes withthe binding to the viral promoter (16), whereas TRIM19/PML interacts with Sp1, also resulting in the inhibition of its transcriptional activity (46). We can thus speculate on TRIM19/PML, and TRIM22 working in a concert to heavily impinge upon the basal transcription of the HIV-1 LTR.

Taken together, these results extend our knowledge on the complex biological mechanisms at the basis of what appears more and more as a “concerted action” of TRIM22, TRIM19/PML, and CIITA RF against HIV-1 viral replication. By favoring the co-localization of CIITA, CyclinT1, and TRIM19/PML in these newly defined TRIM22-containing nuclear bodies, both the basal and the Tat-dependent HIV-1 transcription are inhibited, highlighting a better strategy to counteract virus replication and spreading.

## Author Contributions

GF, GT, and RA participated in the conception and design of the study, performed experiments, analyzed data, and wrote the manuscript. FT participated in the conception and design of the study, performed experiments, analyzed data, and revised the manuscript. GP and EV participated in the conception and design of the study, assisted in experiments and data analysis, and revised the manuscript. All the authors read, critiqued, and approved the final manuscript.

## Disclaimer

The funders had no role in study design, data collection and analysis, decision to publish, or preparation of the manuscript. Funding agencies did not have any role in conducting the study and/or in the preparation of the manuscript.

## Conflict of Interest Statement

The authors declare that the research was conducted in the absence of any commercial or financial relationships that could be construed as a potential conflict of interest.

## References

[1] Neil S Bieniasz P. Human Immunodeficiency virus, restriction factors, and interferon. J Interferon Cytokine Res (2009) 29(9):569–78. 10.1089/jir.2009.0077 19694548 PMC2956573

[2] Jia X Zhao Q Xiong Y HIV suppression by host factors and viral immune evasion. Curr Opin Struct Biol (2015) 31:106–14. 10.1016/j.sbi.2015.04.004 25939065 PMC4476947

[3] Ozato K Shin DM Chang TH Morse HC III. Trim family proteins and their emerging roles in innate immunity. Nat Rev Immunol (2008) 8(11):849–60. 10.1038/nri2413 18836477 PMC3433745

[4] Turrini F Di Pietro A Vicenzi E. Lentiviral effector pathways of TRIM proteins. DNA Cell Biol (2014) 33(4):191–7. 10.1089/dna.2014.2374 24611907

[5] Hattlmann CJ Kelly JN Barr SD. TRIM22: a diverse and dynamic antiviral protein. Mol Biol Int (2012) 2012:153415. 10.1155/2012/153415 22649727 PMC3356915

[6] Lukic Z Hausmann S Sebastian S Rucci J Sastri J Robia SL TRIM5a associates with proteasomal subunits in cells while in complex with HIV-1 virions. Retrovirology (2011) 8:93 10.1186/1742-4690-8-93 22078707 PMC3279310

[7] Pertel T Hausmann S Morger D Zuger S Guerra J Lascano J TRIM5 is an innate immune sensor for the retrovirus capsid lattice. Nature (2011) 472:361–5. 10.1038/nature09976 21512573 PMC3081621

[8] Bernardi R Pandolfi PP Structure, dynamics and function of promyelocytic leukemia nuclear bodies. Nat Rev Mol Cell Biol (2007) 8:1006–16. 10.1038/nrm2277 17928811

[9] Everett RD Chelbi-Alix MK. PML and PML nuclear bodies: implications in antiviral defence. Biochimie (2007) 89:819–30. 10.1016/j.biochi.2007.01.004 17343971

[10] Tavalai N Stamminger T New insights into the role of subnuclear structure ND10 for viral infection. Biochim Biophys Acta (2008) 1783:2207–21. 10.1016/j.bbamcr.2008.08.004 18775455

[11] Lusic M Marini B Ali H Lucic B Luzzati R Giacca M. Proximity to PML nuclear bodies regulates HIV-1 latency in CD4+ T cells. Cell Host Microbe (2013) 13:665–77. 10.1016/j.chom.2013.05.006 23768491

[12] Masroori N Merindol N Berthoux L. The interferon-induced antiviral protein PML (TRIM19) promotes the restriction and transcriptional silencing of lentiviruses in a context-specific, isoform-specific fashion. Retrovirology (2016) 13:19. 10.1186/s12977-016-0253-1 27000403 PMC4802722

[13] Barr SD Smiley JR Bushman FD. The interferon response inhibits HIV particle production by induction of TRIM22. PLoS Pathog (2008) 4(2):e000007. 10.1371/journal.ppat.1000007 18389079 PMC2279259

[14] Tissot C Mechti N. Molecular cloning of a new interferon-induced factor that represses human immunodeficiency virus type 1 long terminal repeat expression. J Biol Chem (1995) 270:14891–8. 10.1074/jbc.270.25.14891 7797467

[15] Kajaste-Rudnitski A Marelli SS Pultrone C Pertel T Uchil PD Uchil PD TRIM22 inhibits HIV-1 transcription independently of its E3 ubiquitin ligase activity, Tat, and NF-kB-responsive long terminal repeat elements. J Virol (2011) 85:5183–96. 10.1128/JVI.02302-10 21345949 PMC3126207

[16] Turrini F Marelli S Kajaste-Rudnitski A Lusic M Van Lint C Das AT HIV-1 transcriptional silencing caused by TRIM22 inhibition of Sp1 binding to the viral promoter. Retrovirology (2015) 12(1):104 10.1186/s12977-015-0230-0 26683615 PMC4683785

[17] Accolla RS Jotterand-Bellomo M Scarpellino L Maffei A Carra G Guardiola J. Air-1, a newly found locus on mouse chromosome 16 encoding a trans-acting activator factor for MHC class II gene expression. J Exp Med (1986) 164:369–74. 10.1084/jem.164.1.369 3088202 PMC2188193

[18] Steimle V Otten LA Zufferey M Mach B Complementation cloning of an MHC class II transactivator mutated in hereditary MHC class II deficiency (or bare lymphocyte syndrome). Cell (1993) 75:135–46. 10.1016/S0092-8674(05)80090-X 8402893

[19] Fontes JD Kanazawa S Nekrep N Peterlin BM The class II transactivator CIITA is a transcriptional integrator. Microbes Infect (1999) 1:863–9. 10.1016/S1286-4579(99)00232-4 10614003

[20] Accolla RS Mazza S De Lerma Barbaro A De Maria A Tosi G. The HLA class II transcriptional activator blocks the function of HIV-1 Tat and inhibits viral replication. Eur J Immunol (2002) 32:2783–91. 10.1002/1521-4141(2002010)32:10<2783::AID-IMMU2783>3.0.CO;2-E 12355430

[21] Tosi G Pilotti E Mortara L De Lerma Barbaro A Casoli C Accolla RS. Inhibition of human T cell leukemia virus type 2 replication by the suppressive action of class II transactivator and nuclear factor Y. Proc Natl Acad Sci U S A (2006) 103:12861–6. 10.1073/pnas.0601589103 16908858 PMC1568938

[22] Casoli C De Lerma Barbaro A Pilotti E Bertazzoni U Tosi G Accolla RS The MHC class II transcriptional activator (CIITA) inhibits HTLV-2 viral replication by blocking the function of the viral trans-activator Tax-2. Blood (2004) 103:995–1001. 10.1073/pnas.0601589103 14525769

[23] Tosi G Forlani G Andresen V Turci M Bertazzoni U Franchini G Major histocompatibility complex class II transactivator CIITA is a viral restriction factor that targets human T-cell lymphotropic virus type 1 Tax-1 function and inhibits viral replication. J Virol (2011) 85(20):10719–29. 10.1128/JVI.00813-11 21813598 PMC3187506

[24] Orlandi C Forlani G Tosi G Accolla RS. Molecular and cellular correlates of the CIITA-mediated inhibition of HTLV-2 tax-2 transactivator function resulting in loss of viral replication. J Transl Med (2011) 9:106. 10.1186/1479-5876-9-106 21736733 PMC3141499

[25] Forlani G Abdallah R Accolla R Tosi G. The MHC-II transactivator CIITA, a restriction factor against oncogenic HTLV-1 and HTLV-2 retroviruses: similarities and differences in the inhibition of Tax-1 and Tax-2 viral transactivators. Front Microbiol (2013) 4:234. 10.3389/fmicb.2013.00234 23986750 PMC3749491

[26] Forlani G Turrini F Ghezzi S Tedeschi A Poli G Accolla RS The MHC-II transactivator CIITA inhibits Tat function and HIV-1 replication in human myeloid cells. J Transl Med (2016) 14:94. 10.1186/s12967-016-0853-5 27089879 PMC4835826

[27] Sahin U Lallemand-Breitenbach V de Thé H. PML nuclear bodies: regulation, function and therapeutic perspectives. J Pathol (2014) 234(3):289–91. 10.1002/path.4426 25138686

[28] Tosi G Jabrane-Ferrat N Peterlin BM. Phosphorylation of CIITA directs its oligomerization, accumulation and increased activity on MHCII promoters. EMBO J (2002) 21(20):5467–76. 10.1093/emboj/cdf557 12374747 PMC129089

[29] Raval GU Bidoia C Forlani G Tosi G Gessain A Accolla RS. Localization, quantification and interaction with host factors of endogenous HTLV-1 HBZ protein in infected cells and ATL. Retrovirology (2015) 12:59. 10.1186/s12977-015-0186-0 26140924 PMC4491271

[30] Negorev D Maul GG Cellular proteins localized at and interacting with ND10/PML nuclear bodies/PODs suggest functions of a nuclear depot. Oncogene (2001) 20:1633–40. 10.1038/sj.onc.1204764 11704851

[31] Ulbricht T Alzrigat M Horch A Reuter N von Mikecz A Steimle V PML promotes MHC class II gene expression by stabilizing the class II trasactivator. J Cell Biol (2012) 199:49–63. 10.1083/jcb.201112015 23007646 PMC3461510

[32] Geoffroy MC Jaffray EG Walker K Hay RT. Arsenic-induced SUMO-dependent recruitment of RNF4 into PML nuclear bodies. Mol Biol Cell (2010) 21:4227–39. 10.1091/mbc.E10-05-0449 20943951 PMC2993750

[33] Lallemand-Breitenbach V Jeanne M Benhenda S Nasr R Lei M Peres L Arsenic degrades PML or PML-RAR alpha through a SUMO-triggered RNF4/ubiquitin-mediated pathway. Nat Cell Biol (2008) 10:547–55. 10.1038/ncb1717 18408733

[34] Hands KJ Cuchet-Lourenco D Everett RD Hay RT. PML isoforms in response to arsenic: high-resolution analysis of PML body structure and degradation. J Cell Sci (2014) 127:365–75. 10.1242/jcs.132290 24190887 PMC3889398

[35] Marcello A Ferrari A Pellegrini V Pegoraro G Lusic M Beltram F Recruitment of human cyclin T1 to nuclear bodies through direct interaction with the PML protein. EMBO J (2003) 22:2156–66. 10.1093/emboj/cdg205 12727882 PMC156077

[36] Garber ME Jones KA. HIV-1 Tat: coping with negative elongation factors. Curr Opin Immunol (1999) 11:460–5. 10.1016/S0952-7915(99)80077-6 10448148

[37] Goffinet C. Cellular antiviral factors that target particle infectivity of HIV-1. Curr HIV Res (2016) 14(3):211–6. 10.2174/1570162X14666151216145521 26674651 PMC5403965

[38] Merindol N Berthoux L. Restriction factors in HIV-1 disease progression. Curr HIV Res (2015) 13(6):448–61. 10.2174/1570162X13666150608104412 26051387

[39] Sivaramakrishnan G Sun Y Rajmohan R Lin VCL. B30.2/SPRY domain in tripartite motif-containing 22 is essential for the formation of distinct nuclear bodies. FEBS Lett (2009) 583(12):2093–9. 10.1016/j.febslet.2009.05.036 19481078

[40] Sivaramakrishnan G Sun Y Tan SK Lin VCL. Dynamic localization of tripartite motif-containing 22 in nuclear and nucleolar bodies. Exp Cell Res (2009) 315(8):1521–32. 10.1016/j.yexcr.2009.01.028 19331816

[41] Gao B Duan Z Xu W Xiong S. Tripartite motif-containing 22 inhibits the activity of hepatitis b virus core promoter, which is dependent on nuclear-located RING domain. Hepathology (2009) 50(2):424–33. 10.1002/hep.23011 19585648

[42] Yu S Gao B Duan Z Xu W Xiong S Identification of tripartite motif-containing 22 (TRIM22) as a novel NF-kB activator. Biochem Biophys Res Commun (2011) 410(2):247–51. 10.1016/j.bbrc.2011.05.124 21651891

[43] Petersson J Lonnbro P Herr AM Morgelin M Gullberg U Drott K The human IFN-inducible p53 target gene TRIM22 colocalized with the centrosome independently of cell cycle phase. Exp Cell Res (2010) 316(4):568–79. 10.1016/j.yexcr.2009.12.007 20006605

[44] Guan D Kao HY The function, regulation and therapeutic implications of the tumor suppressor protein, PML. Cell Biosci (2015) 5:60 10.1186/s13578-015-0051-9 26539288 PMC4632682

[45] Barboric M Nissen RM Kanazawa S Jabrane-Ferrat N Peterlin BM NF-kB binds P-TEFb to stimulate transcriptional elongation by RNA polymerase II. Mol Cell (2001) 8:327–37. 10.1016/S1097-2765(01)00314-8 11545735

[46] Li J Zou WX Chang KS Inhibition of Sp-1 functions by its sequestration into PML nuclear bodies. PLoS One (2014) 9(4):e94450 10.1371/journal.pone.0094450 24728382 PMC3984170

